# Treatment of Diphtheria

**Published:** 1879-01

**Authors:** 


### The Treatment of Diphtheria.

Paris, as our readers are aware, has been, during the last two years, the seat of a formidable epidemic of diphtheria, and the continued prevalence of the disease during the end of the last and the commencement of this year has led to the publication of the experience of several physicians regarding its treatment. Their statements have been summarised by Dr. Ferrand in a recent number of *L' Union Medicale.* It is hardly necessary to say that none of the recorded experience is in favor of the notion of a specific remedy, but the characteristic of the therapeutics of the present day, the use of antiseptics, is strongly marked in the recommended practice. Antiseptic applications have here one of the most obvious and simple of their fields, on account of the nature, constancy, and accessibility of the peculiar anatomical features of the disease. Tannin was employed as a local application by Trousseau, and its use is a somewhat novel fashion has been recommended by Dr. Créquy. He employs inhalations of steam from boiling water in which tannin is suspended. The vapor of the water carries with it a considerable quautity of tannin, although whether enough to exercise an appreciable influence on the exudation is somewhat doubtful. Salicylate of soda has been given internally by M. Cadet de Gassicourt, but without any satisfactory results, and salicylic acid was equally useless in the hands of Dr. Bergeron. Carbolic camphor has been praised by Dr. Soulez. This is a solution of twenty-five grammes of powdered camphor dissolved in one gramme of alcohol and nine grammes of carbolic acid. It is employed pure, or mixed with oil of almonds, as a local application to the false membrane, which is said rapidly to separate and disappear, leaving behind it a simple ulceration which quickly heals. No remedy, however, has received so much commendation as an old one—chlorate of potash. It has been strongly recommended in the past by many authorities, especially in Paris by Isambert, and Seeligmuller has lately insisted afresh on its value. He gives it in solution at least every hour, and pins his faith upon it so exclusively as to consider all other treatment necessary only for their "useful moral effect upon the parents." The removal of the fetor, the gradual disappearance of the false membranes, the reparation of ulceration, and rapid improvement in the general state, are the effects attributed by Seeligmuller to chlorate of potash. This last effect has been ascribed to a fancied power of carrying oxygen to the blood, which contains but little, in consequence of the action on it of the diphtheric bacteria. Its utility is corroborated by Ferrand, who, however, doubts its influence on the general state of the patients, and ascribes its action rather to its excretion by the salivary and pharyngeal glands. He has observed sometimes that the stomach does not bear it well, and even rejects it constantly. Bucquoy and Blache also recommend it. Cadet de Gassicourt made a comparative trial of chlorate of potash, cubebs, and salicylate of soda, at the Hospital Ste. Eugênie, and does not hesitate to accord the first place to the chlorate. Cubebs and copaiba have, however, found an advocate in Trideau, who recommends, to reduce the difficulties of administration to a minimum, that they should be masked with sugar and Malagar wine. Tincture of eucalyptus was employed and recommended by Walcker; it was made by diluting ten grammes of alcoholic extract of eucalyptus by thirty-eight grammes of syrup, and was given after an emetic. All these agents thus act chiefly by modifying the affected surface, and their supposed action might be termed a "local alteration." Actual caustics have of late fallen into disrepute, although nitrate of silver is still recommended by Guillon as an application not only to, but around, the false membranes; a method deprecated by Ferrand, on the theoretical ground that the irritated surfaces become the seat of fresh membrane, like a similar surface elsewhere; and he joins Dr. Rose Cormack in advocating the simplest local application—glycerine of borax or a weak solution of hydrochloric acid, lime-water or sulphate of soda; and he recommends the alternation of these very inoffensive application.—*Lancet.*

